# Identification of hub genes associated with osteoporosis development by comprehensive bioinformatics analysis

**DOI:** 10.3389/fgene.2023.1028681

**Published:** 2023-02-23

**Authors:** Yuxuan Deng, Yunyun Wang, Qing Shi, Yanxia Jiang

**Affiliations:** ^1^ Department of Endocrinology, First Affiliated Hospital of Nanchang University, Nanchang, China; ^2^ Academic Affairs Office, The First Affiliated Hospital of Nanchang University, Nanchang, China

**Keywords:** osteoporosis, WGCNA, PPI network, GSEA analysis, GEO

## Abstract

Osteoporosis (OP) is a systemic bone disease caused by various factors, including, the decrease of bone density and quality, the destruction of bone microstructure, and the increase of bone fragility. It is a disease with a high incidence in a large proportion of the world’s elderly population. However, osteoporosis lacks obvious symptoms and sensitive biomarkers. Therefore, it is extremely urgent to discover and identify disease-related biomarkers for early clinical diagnosis and effective intervention for osteoporosis. In our study, the Linear Models for Microarray Data (LIMMA) tool was used to screen differential expressed genes from transcriptome sequencing data of OP blood samples downloaded from the GEO database, and cluster Profiler was used for enriching analysis of differently expressed genes. In order to analyzed the relevance of gene modules, clinical symptoms, and the most related module setting genes associated with disease progression, we adapted Weighted Gene Co-expression Network Analysis (WGCNA) to screen and analyze the related pathways and relevant molecules. We used the Search Tool for the Retrieval of Interacting Genes/Proteins (STRING) database to construct protein interaction network of key modules, and Cytoscape software was used to complete network visualization and screen of core genes in the network. Various plug-in algorithms of cytoHubba were used to identify key genes of OP. Finally, correlation analysis and single-gene gene probe concentration analysis (GSEA) analysis were performed for each core gene. Results of a total of 8 key genes that were closely related to the occurrence and development of OP were screened out, which provided a brand-new idea for the clinical diagnosis and early prevention of OP. Quantitative real-time PCR (qRT-PCR) was performed for validation, the expression levels of CUL1, PTEN and STAT1 genes in the OS group were significantly higher than in the non-OS groups. Receiver operating characteristic analysis demonstrated that CUL1, PTEN and STAT1 displayed considerable diagnostic accuracy for OS.

## Introduction

Osteoporosis (OP) is a type of orthopedic disease with bone fragility and fracture risk increase due to the decrease of bone density (Lane, 2006; Singanayagam et al., 2021). Statistics demonstrated that the incidence of OP and osteoporotic fractures increased significantly (Armas and Recker, 2012; Demontiero et al., 2012). The prevalence of osteoporosis in the world was reported to be 18.3 (95% CI 16.2–20.7) (Salari et al., 2021). The parts of bones most commonly affected by osteoporosis and osteoporotic fractures include the lumbar and thoracic spine, the distal radius, the proximal femur and the humerus (Yavropoulou et al., 2019). Additionally, among them, vertebral fractures are often accompanied by acute or chronic pain, which lower the quality of life and shorten the lifespan (Marini et al., 2019). Osteoporotic fractures include hip fractures have the highest morbidity and mortality out of all osteoporotic fractures, and they are the most common cause of disability in the elderly (Chandra et al., 2018; Long et al., 2020). The assessments of the European Union (EU) illustrated that as life expectancy increased, the economic burden of osteoporotic fractures was anticipated to increase by an average of 25% by 2025 (Svedbom et al., 2013). Osteoporosis is normally only discovered after patients experience a fracture, which is due to the lack of obvious symptoms and sensitive biomarkers (Sanchez-Riera et al., 2014; Tarrant and Balogh, 2020). Early diagnosis and timely intervention are conducive to hindering the malignant development of OP. Therefore, it is important to find and identify specific and sensitive biomarkers for diagnosis and treatments of OP.

In recent years, a variety of bioinformatics methods have been widely used in the analysis of OP-related potential biomarkers, and multiple genes related to the occurrence and prognosis of OP have been discovered, including peptidylprolyl isomerase domain and WD repeat containing 1 (PPWD1) (Qian et al., 2019) and estrogen receptor 1 gene (Mondockova et al., 2018), which can be used as potential markers for OP diagnosis. Weighted correlation network analysis, also known as weighted gene co-expression network analysis (WGCNA), has been widely used in genomics and in interpreting the expression patterns of disease transcriptomes (Zhao et al., 2010; Pei et al., 2017). Our study collected transcriptome sequencing data of different OP blood samples from the GEO database and used WGCNA to screen and enrich the gene modules that related to the occurrence and development of OP. The study used the STRING database to analyze the protein interaction network of key modules. Finally, we screened out 8 core genes for correlation and single-gene GSEA analysis, which laid the foundation for the early diagnosis of OP and had certain practical values.

## Methods

### Data collection

The mRNA expression profile microarray data were obtained from the GEO dataset: GSE7158 series included 12 OP (low bone density) samples and 14 NC (high bone density) samples; GSE56814 series included 31 pre-treatment samples (15 OP and 16 NC) and 43 prognostic samples, and GSE56815 series included 40 pre-treatment samples (20 OP and 20 NC) and 40 post-treatment samples. For GSE56814 and GSE56815, sample data before treatment were used for difference analysis. As this study involves only a bioinformatics analysis of the GEO data set, no ethical approval was required. The inclusion criteria included age >18 years, osteoporosis fractures grades 1–4 according to OF classification, pathological fractures: osteoporotic fractures, fracture of at least 1 vertebral body, fractures of thoracic or lumbar vertebral body. The exclusion criteria included pathological neoplastic fractures, osteoporosis fractures (OF) grade 5 according to OF classification and AO type B and C fractures.

### Functional enrichment analysis

We carried out functional enrichment analysis of DEGs by analyzing Gene Ontology (GO) terms and Kyoto Encyclopedia of Genes and Genomes (KEGG) pathways by using the cluster profiler package in R (version 4.2.0) (Yu et al., 2012). (*p* < 0.05 is considered statistically significant).

### Weighted gene co-expression network analysis

WGCNA was an algorithm that was used to construct the gene co-expression network, reveal the correlation patterns between genes and provide an explanation for the biological functions of network modules (Langfelder and Horvath, 2008). We screened out the genes that had the significant differences in GSE7158, GSE56814 and GSE56815, and we used WGCNA (version 1.60) to construct the co-expression network.

### PPI network construction and identification of hub genes

We used STRING database (version 11.0) tool to build a protein–protein interaction (PPI) network (Szklarczyk et al., 2019). By using WGCNA (version 1.60), we screened out key module genes and defined them as key genes. After superposition of DEGs and screening key genes, data were put into the STRING database (https://string-db.org/) to construct protein interaction network, and data visualization was performed using Cytoscape (version 3.7.2) (Shannon et al., 2003). Finally, 8 core genes were screened out.

### Analysis by GSEA

GSEA, a kind of enrichment analysis method based on gene sets, has been used to analyze the expressions of genes and select single or multiple MSigDB gene set functions to analyze the correlation of gene expression data and phenotypic genes. Then, based on data expressions of genes and the relevance of phenotypic genes, it was determined which genes in each gene set were enriched in the upper or lower parts of the gene list after the sequencing of the gene. It would help to identify the synergistic changes of effects of genes in this gene set towards phenotypic changes. Based on the results of correlation analysis, Reactome base analysis was performed on the 8 single genes screened. GSEA shows only the top 20 results of each gene (>0, positive correlation between pathway and gene; <0, pathways are negatively correlated with genes).

### Quantitativereal-time RT-PCR (qRT-PCR)

Total RNA was extracted from samples from 10 OS patients and 10 healthy controls using phenol-chloroform (TRIzol; Invitrogen; ThermoFisher Scientific, Inc., Waltham, MA, United States). The quality of RNA was assessed by capillary electrophoresis (Agilent Technologies, Inc., Santa Clara, CA, United States). Libraries for small RNA sequencing were prepared using NEB kits (New England Biolabs, Inc., Ipswich, MA, United States). qRT-PCR with SYBR-Green (Takara, Osaka, Japan) to detect CDC5L, CUL1, CXCL10, EIF2AK2, POLR2B, PTEN, STAT1, and TBP expression levels, GAPDH was applied as a house keeping gene. The reaction was performed *via* 40 amplification cycles using the following protocol: 95°C for 3 min, 95°C for 45 s, 55°C for 15 s, and 72°C for 50 s. Primers used in PCR were shown in Supplementary Table S1. Samples were analyzed in triplicate, and gene expression was quantified by normalizing target gene expression to that of the internal control using the 2^−ΔΔCt^ formula.

### Statistical analysis

Data were analyzed using SPSS 19.0 software. qRT-PCR were repeated three times, and data were represented as the mean ± SD. Two-tailed t-tests were performed to compare the difference in plasma gene expression levels. Student’s t-test was used to determine the significance of difference between two groups. Receiver Operating Characteristic (ROC) curves were performed to determine the diagnostic utility of each selected gene in distinguishing between healthy subjects and OP cases. Estimates of the corresponding area under the curve (AUC) were calculated at the 95% confidence interval (CI). Finally, the cut-off point with the highest specificity and sensitivity was determined.

## Results

### DEGs between low bone mineral density (OP) and high bone mineral density (NC)

**FIGURE 1:**
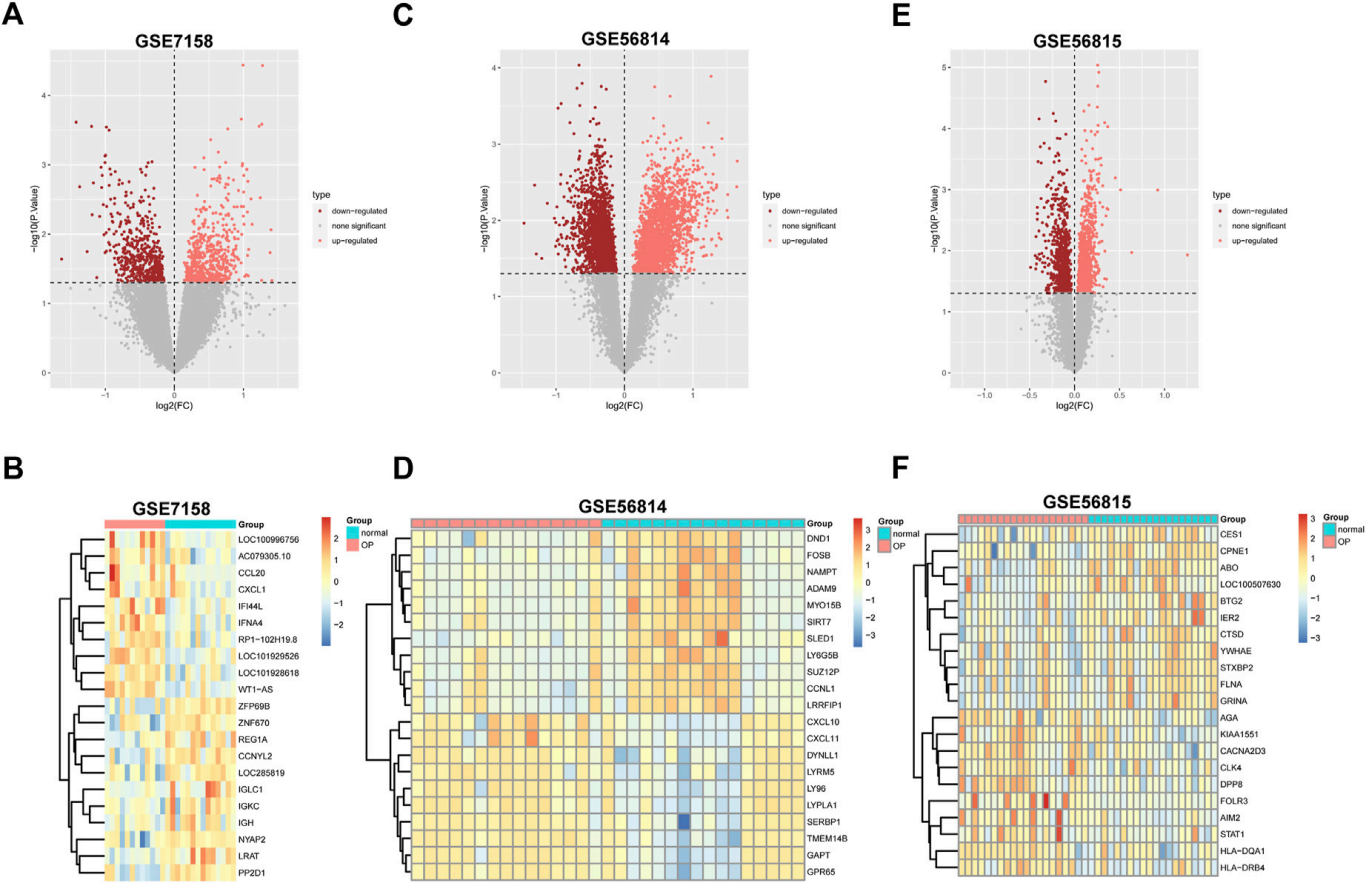
Identification of differentially expressed genes between OP and normal samples. Volcano plots of the differential gene expression data from **(A)** GSE7158, **(C)** GSE56814, and **(E)** GSE56815. In the volcano plots, the pink points show upregulated genes (adjusted *p*-value< 0.05), whereas the red points represent downregulated genes. Heatmap of the top 10 differentially expressed genes based on **(B)** GSE7158, **(D)** GSE56814, and **(F)** GSE56815. The color intensity (from red to blue) suggests the higher to lower expression. FC fold-change.

**FIGURE 2:**
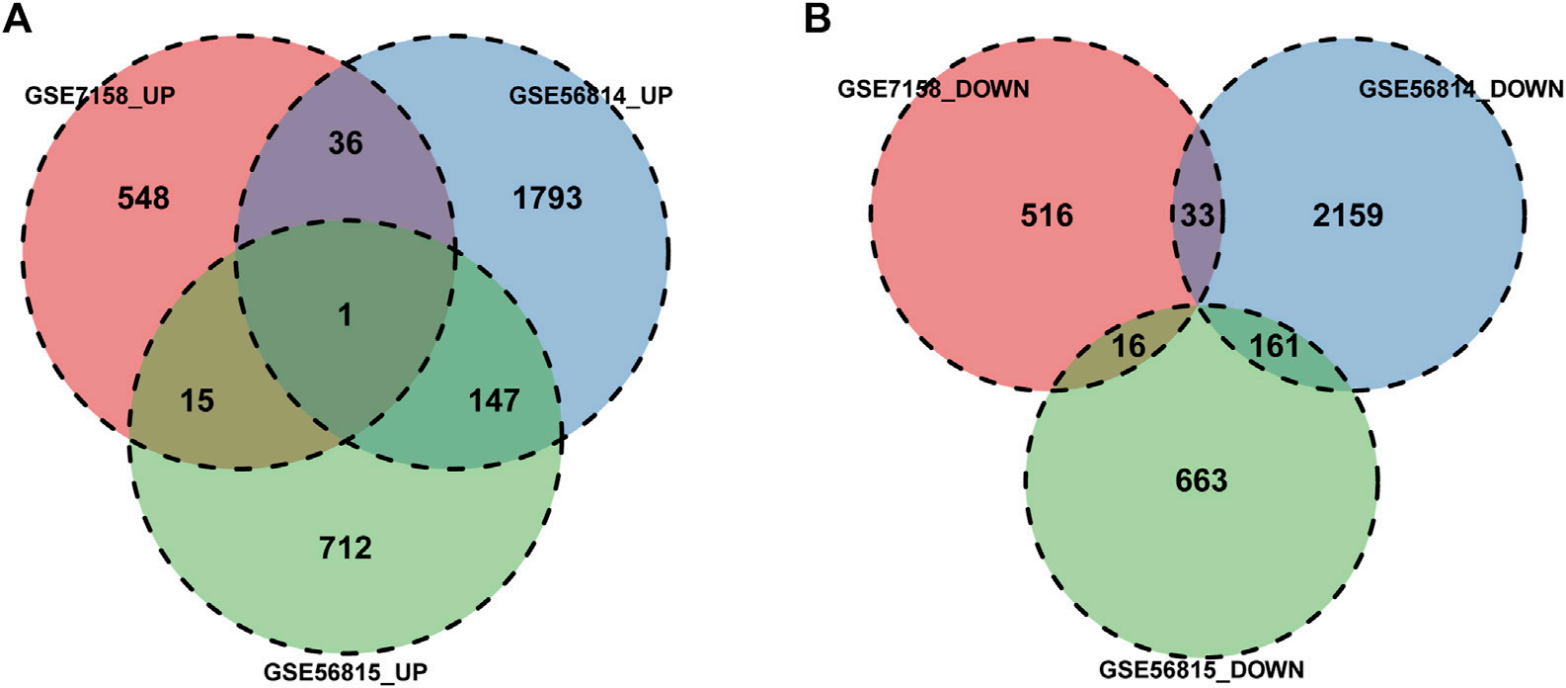
DEGs intersection. **(A)** Differential analysis upregulated gene intersection, **(B)** Differential gene downregulated gene intersection, Genes that were upregulated or downregulated in at least two data sets were selected as differential genes for subsequent analysis.

We used the parameter value of *p* < 0.05, |log(2)FC|>1.5 to preprocess data sets GSE7158, GSE56814 and GSE56815. In GSE7158, we identified 600 up-regulated and 565 down-regulated genes. The volcano diagram for all genes and the expression heatmap of the top 10 DEGs were shown in Figures 1A,B. In GSE56814, we identified 1977 upregulated and 2353 downregulated genes (Figures 1C,D). A total of 875 upregulated genes and 840 downregulated genes were identified in GSE56815 (Figures 1E,F). Subsequently, we conducted gene intersection analysis of upregulated or downregulated genes in different data sets, then screened out genes that were simultaneously upregulated or down-regulated in the two data sets as differential genes for subsequent analysis (Figures 2A,B).

### Differential gene enrichment analysis

**FIGURE 3:**
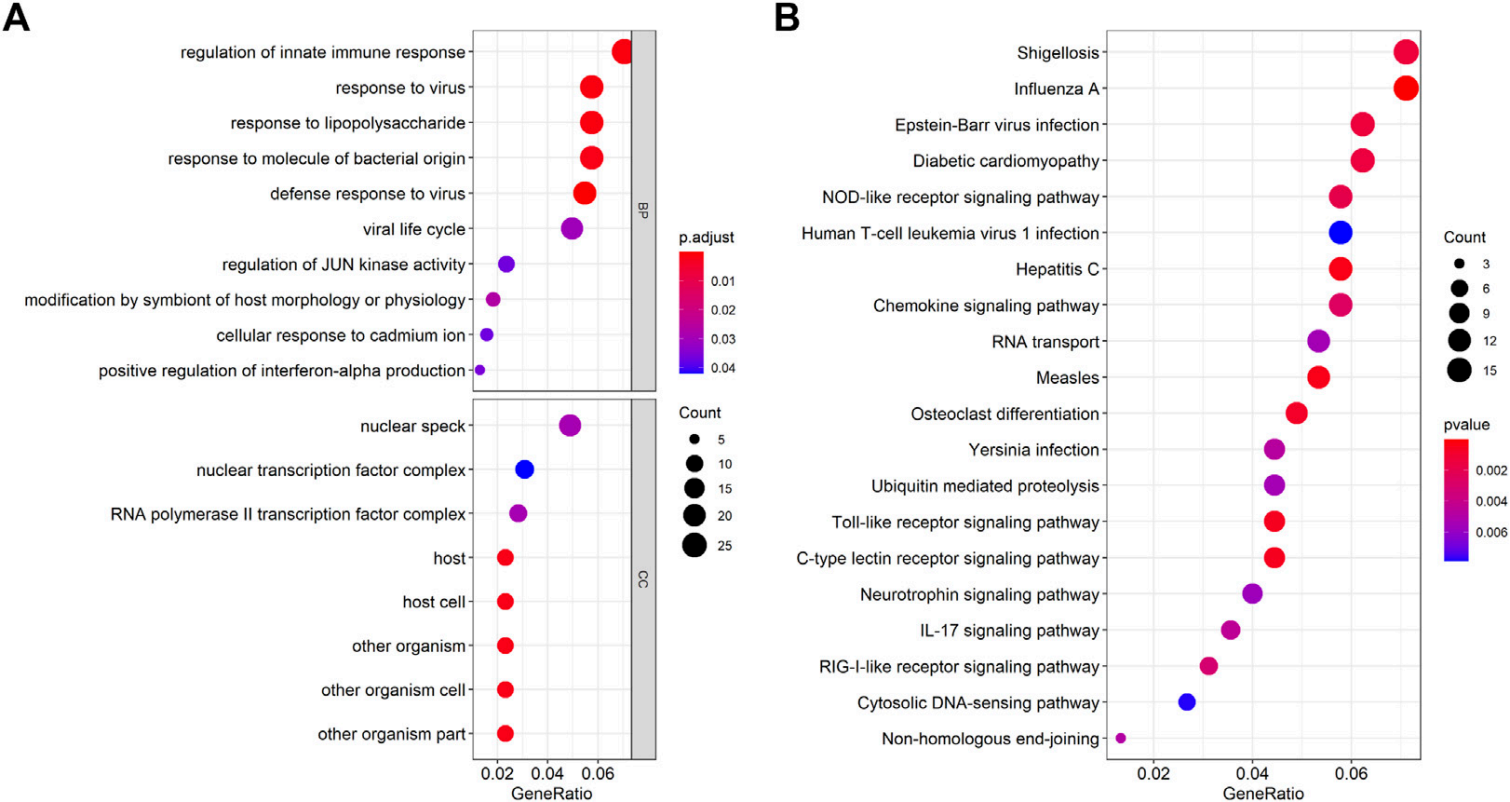
Enrichment analysis of DEGs. **(A)** Enrichment of DEGs using Gene Ontology (GO) analysis, including terms in biological process (BP) and cellular component (CC). **(B)** Kyoto Encyclopedia of Genes and Genomes (KEGG) (right) analysis. The larger the circle in the figure, the more genes it contains; lower *p* values are indicated with a stronger red color.

We conducted enrichment analysis of related molecular functions (MF) (Supplementary Table S1), biological processes (BP) (Supplementary Table S2) and cellular components (CC) (Supplementary Table S3) of genes with stable differences based on the GO and KEGG databases, in which results of MF analysis were not significant (Figure 3A). The biological process of enrichment was mainly related to the immune regulation of the body, and the enrichment of cell components mainly involved nucleus-related molecules. KEGG pathway analysis in Figure 3B showed that the Shigellosis and Influenza A pathways were the most abundant pathways, followed by the Epstein-Barr virus infection, Diabetic cardiomyopathy, and NOD-like receptor signaling pathways, respectively (Supplementary Table S4).

### Construction of co-expression network and key modules identification

**FIGURE 4:**
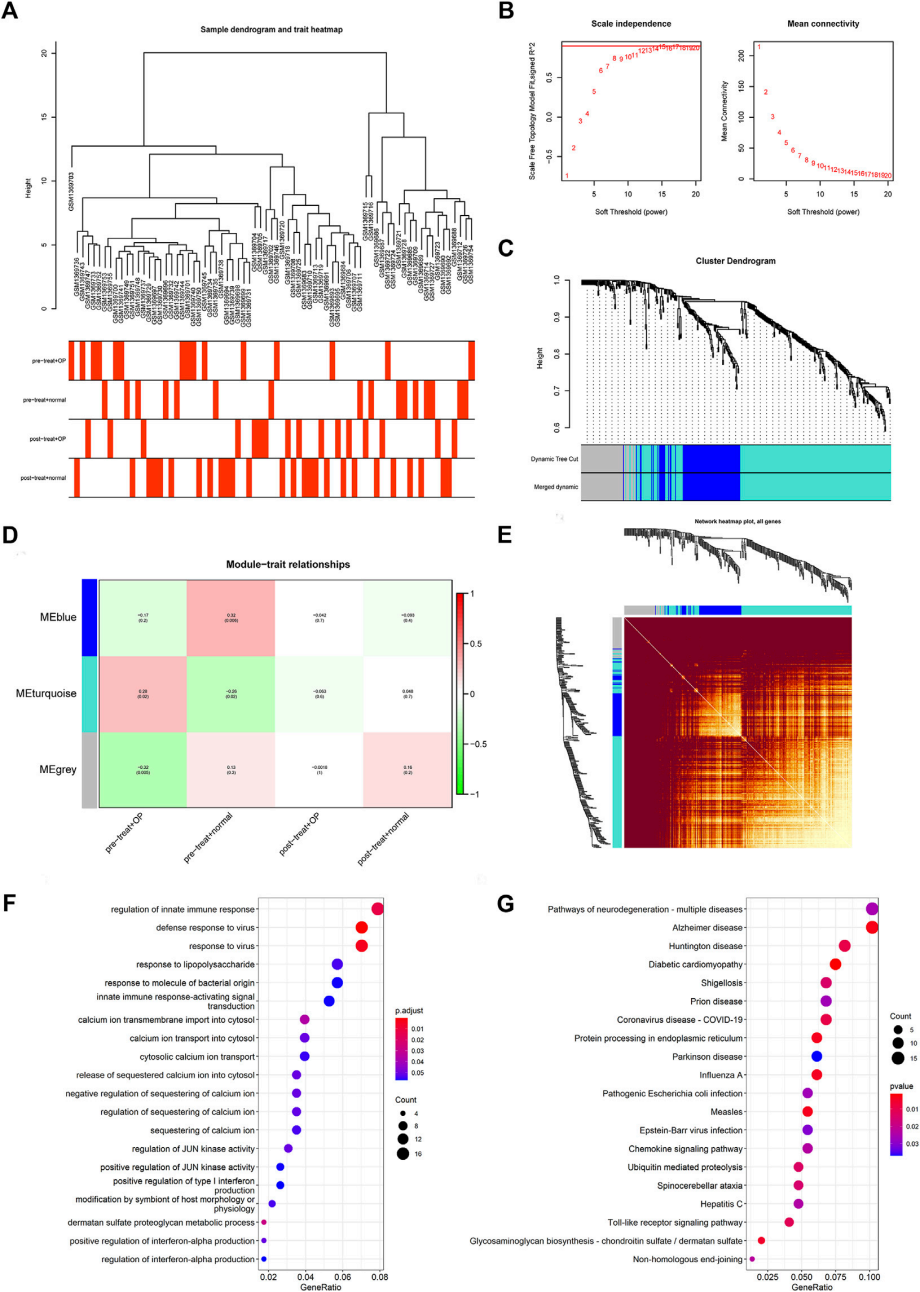
Construction of co-expression network and key modules identification. **(A)** Cluster tree of clinical distribution of samples. Analysis of **(B)** the scale-free fit index and **(C)** the mean connectivity for various soft-thresholding powers. The soft-thresholding power of 14 was selected based on the scale-free topology criterion. **(D)** Correlation between gene modules and clinical traits. Each row corresponds to a module eigengene, each column corresponds to a clinical trait. Each cell contains the corresponding correlation and *p*-value. The table is color-coded by correlation according to the color legend. **(E)** Expression similarity between genes. The higher the similarity, the brighter the color. **(F)** Enrichment BP of DEGs using Gene Ontology (GO) analysis. **(G)** Kyoto Encyclopedia of Genes and Genomes (KEGG) (right) analysis.

The WGCNA software package was used to analyze the intersection genes in the GSE56814 data set, and the clinical shape distribution tree of samples was obtained (Figure 4A). Then, the power value was checked and the optimal result was obtained when the soft-thresholding power was 14. The clustering tree was used to characterize the result (Figures 4B,C).

Using clinical data in the GEO database, we analyzed co-expression modules that related to specific characteristics in OP patients and general control group before and after treatment, then we obtained ME turquoise, which was positively correlated with the occurrence and development of OP for subsequent analysis (Figure 4D). The heat map showed that the similarity of expressions for all genes in the analysis: the higher the intervene similarity, the darker the module color (Figure 4E). In addition, using the GO and KEGG databases, we found that the main biological processes of these genes were concentrated around regulation of innate immune response. For the defense response to the virus, the relevant pathways focused on neurodegenerative polypathies (Figures 4F,G).

### PPI network construction and identification of hub genes

**FIGURE 5:**
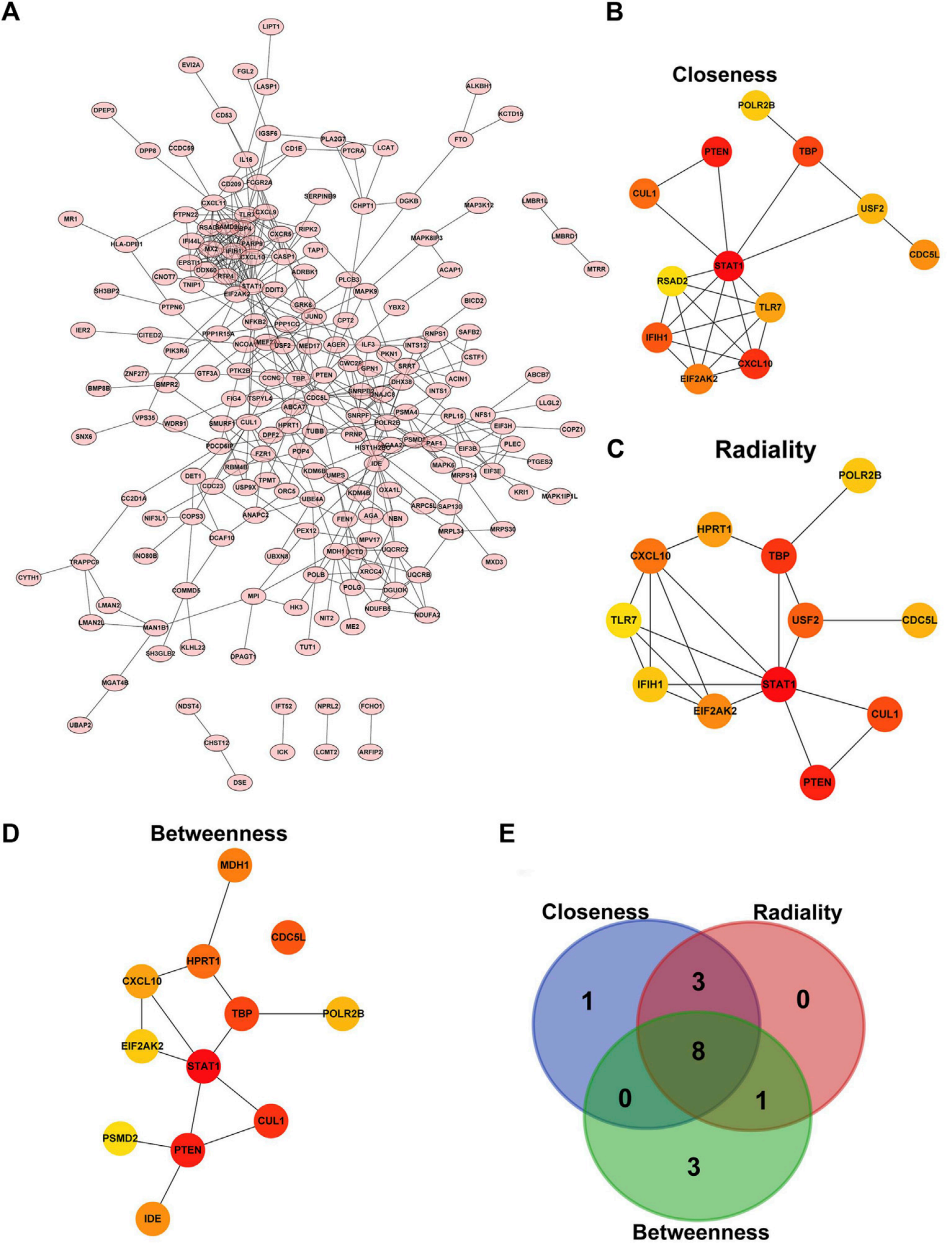
PPI network construction and identification of hub genes. **(A)** The protein–protein interaction network of the overlapped genes. **(B)** CytoHubba plug-in Closeness algorithm identified top12 core genes. **(C)** CytoHubba plug-in Radiality algorithm identified top12 core genes. **(D)** CytoHubba plug-in Betweenness algorithm identified top12 core genes. **(E)** The intersection of core genes screened by three algorithms.

The PPI network constructed by the ME turquoise module gene, which was based on the STRING database, was shown in Figure 5A. In addition, three different algorithms in the cytoHubba plug-in, Closeness, Radiality and Betweenness, were used to identify Top 12 Hub genes in the interaction network, and eight core genes (CDC5L, CUL1, CXCL10, EIF2AK2, POLR2B, PTEN, STAT1, TBP) were screened out by the three algorithms. These genes were selected to conduct follow-up analysis (Figures 5B–E).

### Correlation analysis and differential expression verification of hub gene

**FIGURE 6:**
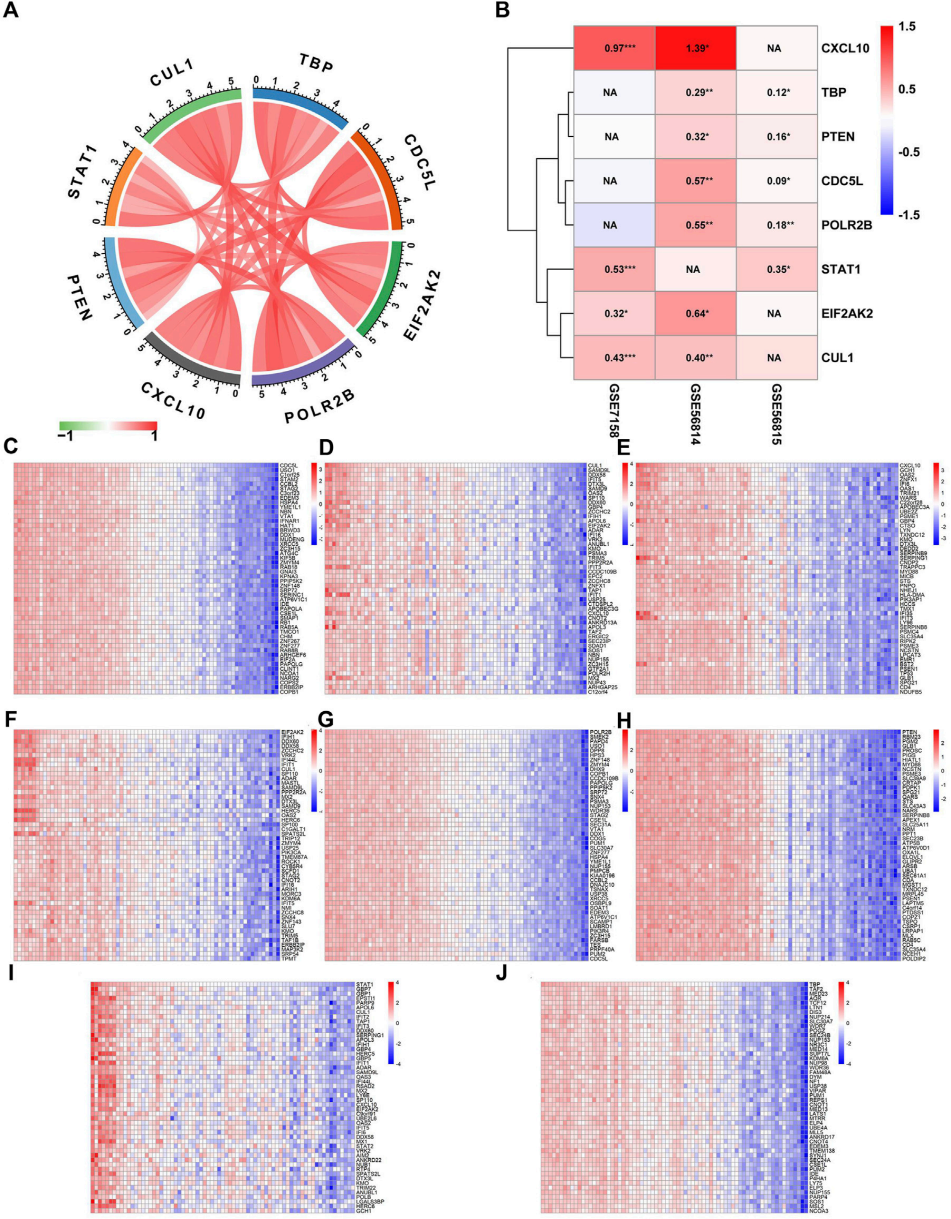
Correlation analysis and differential expression verification of Hub gene. **(A)** Gene correlation analysis. The red line represents a positive correlation, the green line represents a negative correlation, and the darker the color, the stronger the correlation. **(B)** LogFC and *p* values of core genes in different data sets. **(C–J)** Heatmaps of the correlation between core genes and all genes based on data from 74 cases in GSE56814, only the top50 with a positive correlation are shown.

We conducted correlation analyses of 8 core genes in 74 cases of GSE56814 and found that all 8 core genes were positively correlated to OP (Figure 6A). The heat map showed the logFC and *p* values of the 8 core genes in the three data sets (Figure 6B). In addition, the 74 cases of GSE56814 were analyzed for correlation between core genes and all genes, and the top 50 positive correlation genes were obtained as shown in the (Figures 6C–J).

### Single gene GSEA

**FIGURE 7:**
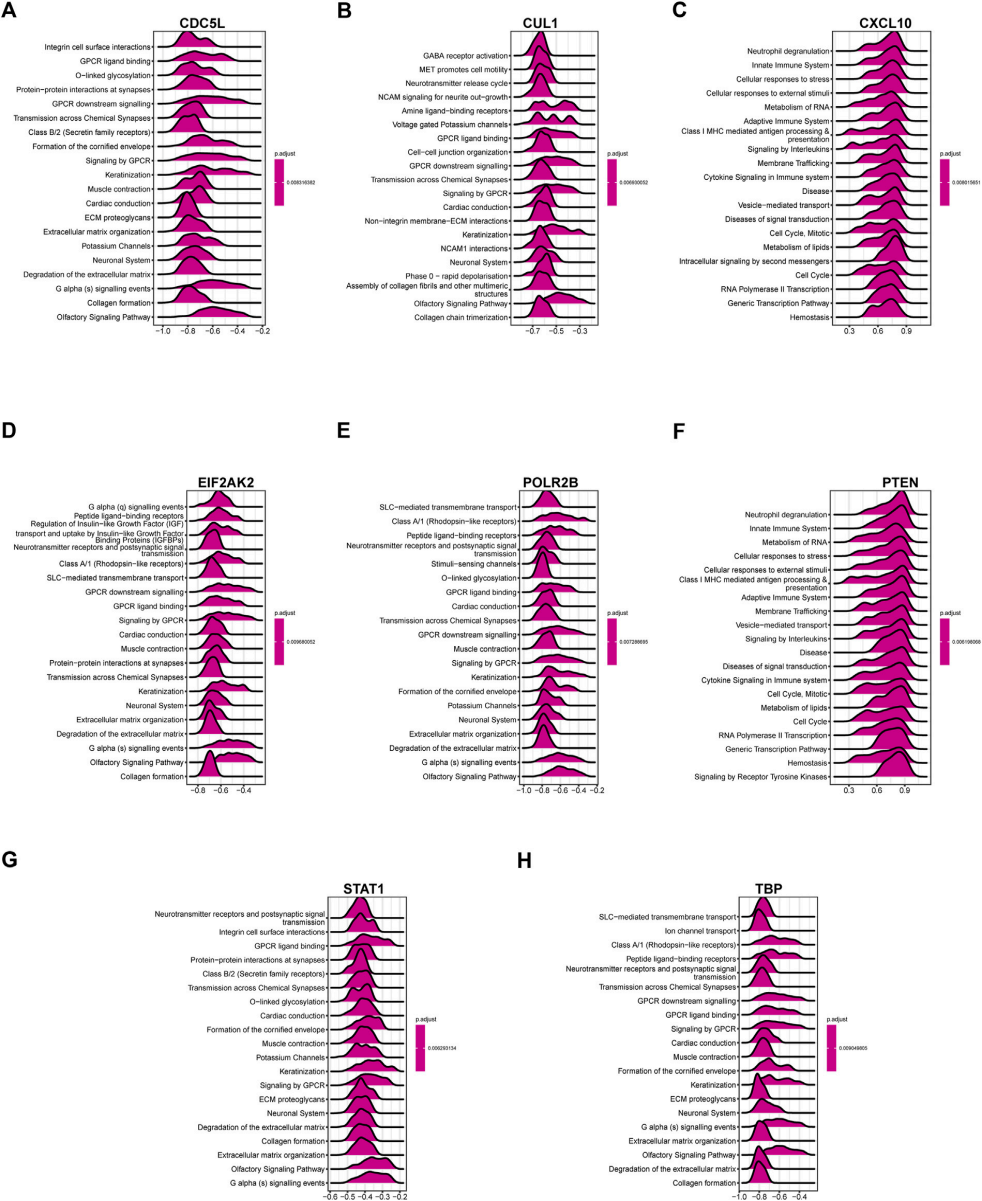
Results of correlation analysis in gene Figure 6 were used for GSEA analysis of single gene. **(A)** CDC5L, **(B)** CUL1, **(C)** CXCL10, **(D)** EIF2AK2, **(E)** POLR2B, **(F)** PTEN, **(G)** STAT1, **(H)** TBP. The top20 results of 8 genes were presented respectively. The abscissa value represents the NES value analyzed by GSEA. If the value is greater than 0, the pathway is positively correlated with the gene; if the value is less than 0, the pathway is negatively correlated with the gene.

Based on the correlation analysis results, we conducted GSEA, which was based on the Reactome algorithm, and the results of top-20 correlation of each gene were shown in Figure 7. The results showed that CDC5L and STAT1 were negatively correlated to integrin cell surface interactions, GPCR ligand binding, and other pathways. The CUL1 pathway was negatively correlated with GABA receptor activation. MET Signaling was negatively correlated with cell motility, and both CXCL10 and PTEN were positively correlated with neutrophil degranulation and innate immune system pathways. EIF2AK2 was inversely related to G alpha(Q) signaling events, peptide ligand binding receptors, and so on. POLR2B and TBP were negatively correlated with SLC-mediated transmembrane transport and Class A/1 (Rhodopsin-like receptors).

### qRT-PCR validation results

**FIGURE 8:**
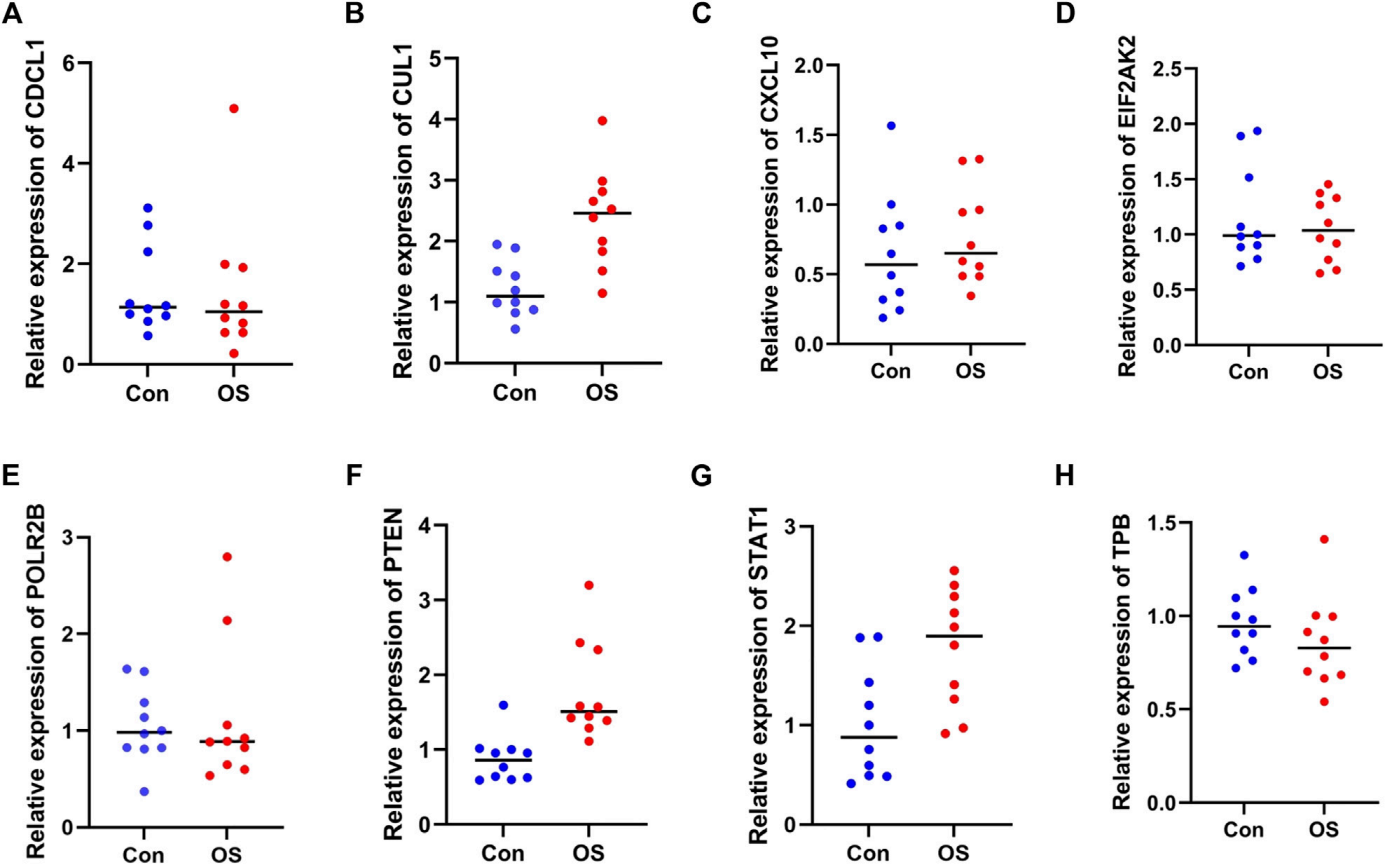
qRT-PCR validation results. **(A)** CDC5L, **(B)** CUL1, **(C)** CXCL10, **(D)** EIF2AK2, **(E)** POLR2B, **(F)** PTEN, **(G)** STAT1, **(H)** TBP. RNA expression of 8 genes were measured in OS and healthy samples. *p*-values were calculated using a two-sided unpaired Student’s t-test. **p < 0.01; ***p < 0.00 1; ****p < 0.000 1.

In the qRT-PCR results, no significant differences were observed in CDC5L, CXCL10, EIF2AK2, POLR2B, TBP (Figure 8). The expression levels of.

CUL1, PTEN and STAT1 gene in the non-OS group were significantly lower than those in the OS group (*p* < 0.05).

### Assessing the diagnostic performances of genes

**FIGURE 9:**
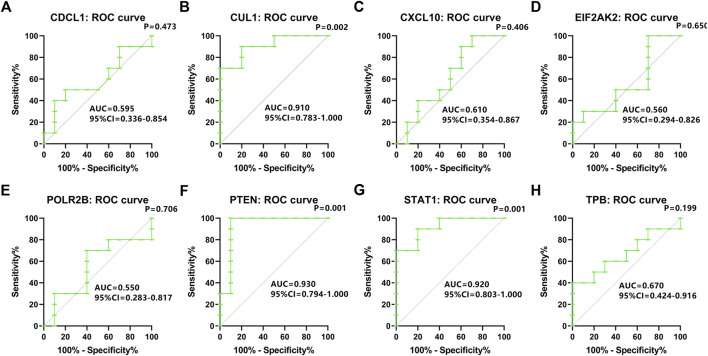
ROC curves. **(A)** CDC5L, **(B)** CUL1, **(C)** CXCL10, **(D)** EIF2AK2, **(E)** POLR2B, **(F)** PTEN, **(G)** STAT1, **(H)** TBP. Diagnostic value of each mRNA to dichotomize healthy subjects and osteoporotic patients.

To evaluate the diagnostic power of the genes found significantly dysregulated during the validation phase, ROC analysis (Figure 9, Panels A–H) was conducted for each subgroup of patients, and the associated area under the curve (AUC) was calculated. CUL1, PTEN, STAT1 exhibited high sensitivity and specificity with AUC values between 0.91 and 0.93 (*p* < 0.01), indicating them as good diagnostic biomarker candidates. In particular, more significant values to distinguish the non-OS group from OS patients were obtained for PTEN, whereas STAT1 resulted more effective to identify OP patients with fracture.

## Discussion

According to the World Health Organization (WHO) reports, OP has become one of the most common diseases in the world, with 30%–50% of women worldwide suffering from fracture due to OP in their lifetime (Rachner et al., 2011). Since OP doesn’t have any obvious clinical symptoms before the occurrence of the first fracture, early diagnosis is key to timely intervention and to alleviating the pain of patients (Dera et al., 2019; Yong and Logan, 2021). Compared to previous studies (Chen et al., 2021), our studies not only screened genes related to OP occurrence and development in the GSE database, but also verified the correlation and differential expressions between core genes and the data set. Furthermore, additional facts confirmed that CDC5L, CUL1, CXCL10, EIF2AK2, POLR2B, PTEN, STAT1, TBP and other 8 genes play an important role in the occurrence of OP. The genes themselves and the signaling pathway marker molecules enriched among them may be potential signal molecules for the early diagnosis of OP. In this study, transcriptome sequencing data of OP blood samples from GEO database were analyzed by using LIMMA algorithm to obtain more than 5,000 groups of differential genes between OP and the control group. After enrichment analysis of differential genes, the WGCNA method was used to screen the gene set modules most related to disease progression, and enrichment was conducted. The results showed that the biological processes that were mainly related to the regulation of innate immune response, response to virus, response to lipopolysaccharide and so on, which indicated that immune regulation plays an important role in osteoporosis development. GO analysis of cell components showed that nuclear speck, nuclear transcription factor complex, RNA polymerase II transcription factor complex were the key components in OP. KEGG pathway analysis showed that Shigellosis, Influenza A and Epstein-Barr virus infection were the significantly changed pathways between the two groups. All the above results of GO analysis and KEGG analysis indicated that immune system dysregulation was found in OP patients. In previous studies, dysregulation of the immune system had been shown to adversely affect bone integrity (McInnes and Schett, 2007). Our results were consistent with results in previous study, and we confirmed that these biological processes related to immune regulation and cellular molecules were closely associated with the occurrence and development of OP.

In our constructed PPI network, cytoHubba analysis was used to obtain 8 key genes related to OP development: CDC5L, CUL1, CXCL10, EIF2AK2, POLR2B, PTEN, STAT1, and TBP. Recent studies had reported the role of CDC5L in bone development. CDC5L was expressed in chondrocytes proliferating from mouse embryonic bone growth plates, and CDC5L mediates the promotion or inhibition of pre-mRNA splicing of early cartilage genes (Sox9 and Col2a1) or Wee1, respectively (Jokoji et al., 2021). CUL1 and CXCL10 were also frequently reported in immune regulation (Sorrentino et al., 2021). Although the functions of other genes had not been confirmed and reported in OP, their roles in other diseases have been demonstrated. For example, STAT1 can affect the development of cervical cancer by regulating the expression of PARP1 (Raspaglio et al., 2021). The studies demonstrated that the 8 genes might be involved in the progression and development of human diseases including OP. Along with the correlation between core genes and the data set, the reverse verification of differential expression, and single-gene GSEA analysis, the studies further confirmed our hypotheses.

Our subsequent research will focus on establishing a clinical diagnostic model based on these 8 keys genes, then on evaluating the usefulness and reliability of the model. Additionally, the unreported role of several other core genes in OP also deserves our attention. However, there was still weakness of our study. For example, the sample size in this study was limited and animal experiments to validate the function of these differential genes are lacking.

In summary, we identified 8 key genes that could be used to establish an early diagnostic model of OP: CDC5L, CUL1, CXCL10, EIF2AK2, POLR2B, PTEN, STAT1, and TBP. The discovery of these genes provided potential molecular targets for the clinical diagnosis and treatment of OP.

## Data Availability

The original contributions presented in the study are included in the article/Supplementary Material, further inquiries can be directed to the corresponding author.
